# Low serum iPTH at the end of surgery is the earliest predictor of postoperative hypocalcemia after total thyroidectomy

**DOI:** 10.1007/s00423-023-03194-8

**Published:** 2023-11-30

**Authors:** Alexandros Lalos, Alexander Wilhelm, Katja Linke, Stephanie Taha-Mehlitz, Beat Müller, Alberto Posabella, Beatrice Kern

**Affiliations:** Clarunis – University Digestive Health Care Center, St. Clara Hospital and University Hospital of Basel, Basel, Switzerland

**Keywords:** Hypocalcemia, Symptomatic hypocalcemia, Hypoparathyroidism, Total thyroidectomy, iPTH, Percentage decrease of iPTH

## Abstract

**Background:**

The most frequent complication of total thyroidectomy remains hypocalcemia due to low postoperative levels of serum intact parathyroid hormone (iPTH). The purpose of this study was to investigate the role of decreased iPTH at the end of surgery in predicting hypocalcemia. In addition, we examined the percentage decrease of iPTH as potential indicator of hypocalcemia.

**Methods:**

We retrospectively collected the data of patients who underwent total thyroidectomy for benign and malignant diseases at our institution between 2010 and 2022. The iPTH level was measured before and at the end of surgery, and serum calcium levels on the first postoperative day. Demographic, clinical, and biochemical characteristics of patients with low iPTH were compared with patients with normal iPTH levels using ANOVA for continuous variables and χ2-tests for categorical variables. Multivariable logistic regression analysis evaluated the association of iPTH at the end of surgery and the relative reduction of iPTH with postoperative hypocalcemia.

**Results:**

The mean age of the 607 patients in this study was 55.6 years, and the female-to-male ratio was 5:1. Goiter was the most common indication for surgery (*N* = 382, 62.9%), followed by Graves’ disease (*N* = 135, 22.2%). The mean preoperative iPTH was 49.0 pg/ml, while the mean postoperative iPTH was 29.3 pg/ml. A total of 197 patients (32.5%) had an iPTH level below normal, 77 patients (39%), had iPTH levels of 10–15.0 pg/ml and 120 patients (61%) of < 10.0 pg/ml at the end of surgery. Among all patients, 124 (20.4%) developed hypocalcemia on the first postoperative day. The mean percentage of decrease of iPTH was highest among patients with iPTH < 10 pg/ml (76.9%, *p* < 0.01); this group of patients had also the highest rate of postoperative hypocalcemia on day one (45.0% vs. 26.0% vs 12.2%, *p* < 0.01).

**Conclusions:**

Measurement of iPTH at the end of total thyroidectomy predicts patients who are at risk for postoperative hypocalcemia. The combination of low serum iPTH with a decrease in iPTH level of ≥ 50% may improve prediction of hypocalcemia compared to iPTH levels alone allowing for early calcium substitution in these patients at high risk of developing postoperative hypocalcemia.

## Introduction

Total thyroidectomy (TT) is among the most commonly performed surgical procedures as it is the preferred treatment for a broad spectrum of thyroid disorders, including both malignant and benign conditions [1]. According to the American Thyroid Association (ATA) and the European Thyroid Association (ETA), differentiated thyroid cancers (DTC) with aggressive features, undifferentiated thyroid cancers, and medullary thyroid cancers require a total thyroidectomy [2–8]. The likelihood of recurrence of benign thyroid disease after subtotal thyroidectomy and the associated difficulty of repeat surgery must be weighed against the higher risk of postoperative complications after total thyroidectomy [9–11].

Despite advancements in the surgical technique and devices, TT is associated with a significantly higher risk of complications compared to thyroid lobectomy even among high-volume surgeons [12]. The parathyroid glands which are responsible for the synthesis of parathyroid hormone – a key player in maintaining calcium balance—are located close to the thyroid capsule. Their position can vary which may result in unintended loss of blood supply, damage, or accidental removal of parathyroid tissue during total thyroidectomy [13, 14]. As a consequence, damage to parathyroid tissue or parathyroid vascularization leads to low postoperative levels of serum intact parathyroid hormone (iPTH). PTH not only enhances the release of calcium from bones into the bloodstream but also decreases the loss of calcium in urine through reabsorption in kidneys. Additionally, it augments the synthesis of 1,25-dihydroxyvitamin D in the kidneys, which is essential for the absorption of calcium from the intestine. Therefore, reduced synthesis of PTH ultimately manifests as hypocalcemia [15, 16]. Low serum calcium levels can cause neuromuscular (paresthesia, tingling, muscle cramps, and spasms) or neuropsychiatric (confusion, irritability, and depressive mood) symptoms [17]. Occasionally, symptoms of hypocalcemia may include potentially life-threatening conditions such as seizures, tetany, arrhythmias, or laryngospasm [18].

Previous studies have already tried to predict postoperative hypocalcemia as early and as accurately as possible. Combined measurement of intact parathyroid hormone (iPTH) and calcium levels after the operation are used worldwide to predict postoperative hypoparathyroidism [19, 20]. Grodski et al. analyzed 27 studies that investigated the postoperative measurement of serum iPTH [21]. The sample was taken after skin closure or at a range from 5 min up to 48 h after surgery. The results demonstrated that post-thyroidectomy PTH levels accurately predict hypocalcemia but lack 100% accuracy, which may be one reason why there is still no standardized method to predict postoperative hypocalcemia.

Since the perfect timing for iPTH measurement is not yet defined in guidelines the aim of this study was to investigate the incidence of low postoperative iPTH at the end of surgery and its reliability in the prediction of hypocalcemia. Detection of patients at risk of developing hypocalcemia as early as possible allows for early calcium supplementation to avoid potential life-threatening complications. Furthermore, we investigated the role of the relative reduction of iPTH as a potential predictor of hypocalcemia.

## Methods

### Study population

In this study we retrospectively analyzed prospectively collected data from patients, who were operated between 2010 and 2022 at the St. Clara Hospital of the Clarunis—University Digestive Health Care Center, St. Clara Hospital and University Hospital of Basel in Switzerland. 607 individuals, who underwent total thyroidectomy with or without central and/or lateral lymph node dissection with complete information on pre- and postoperative laboratory tests were included. Indications for surgery were multinodular goiters, differentiated thyroid cancer (DTC), undifferentiated thyroid carcinoma (UTC), medullary thyroid carcinoma, Graves’ disease, refractory Hashimoto’s disease, non-invasive follicular thyroid neoplasm with papillary-like nuclear features (NIFTP), and lymphoma. Exclusion criteria were surgical procedures other than total thyroidectomy, preoperative disease of the parathyroid glands, and missing values of laboratory analysis. The study was reviewed and approved by our institutional review board (Ethics Committee of Basel, EKBB 2020–01205) and the consent was waived by the ethics committee (Ethikkommission Nordwest- und Zentralschweiz).

### Biochemical measurements and reference ranges

Serum iPTH was measured twice: once before surgery, and again at the end of the operation, at the time of skin closure. The iPTH assay at our hospital has a normal range between 15.0 and 80.0 pg/ml. Serum calcium (Ca) levels were checked in the morning of the first postoperative day. Hypocalcemia was defined as total serum calcium level of < 2.2 mmol/l according to normal ranges at our institution. The percentage decrease of iPTH was calculated using the difference of preoperative iPTH and levels at the end of surgery.

### Operative technique

All patients underwent total thyroidectomy that were performed either by a specialized endocrine consultant or by experienced surgeons supervised by a specialized endocrine consultant. Throughout the procedures, all surgeons employed surgical loupes and made every efforts to visualize and protect the four parathyroid glands and the recurrent laryngeal nerves (RLN). Intraoperative nerve monitoring was used to facilitate identification and protection of the recurrent laryngeal nerves. A parathyroid gland was selectively removed and autotransplanted into the homolateral sternocleidomastoid muscle if its vascularization seemed to be limited or accidentally removed with the thyroid gland. No fluorescence technique to assess the vascularity/functionality of these glands was used.

### Statistical analysis

Demographic, clinical, biochemical, and pathological characteristics of patients with low iPTH at the end of surgery were compared to patients with normal iPTH levels using ANOVA for continuous variables and χ2-tests for categorical variables. Multivariable logistic regression models evaluated the associations of postoperative hypocalcemia and perioperative variables. The statistical fit of three different models was evaluated using Akaike information criterion. Co-variables were chosen according to clinical judgement. All models included patients’ age, sex, American Society of Anesthesia (ASA) score, surgeon volume (high volume defined as > 50 thyroidectomies per year), indication for surgery, extent of surgery (TT vs. TT with central lymph node dissection) as co-variables. The first model further comprised postoperative iPTH levels, the second model percentage decrease of iPTH, and the third model used both iPTH levels and the percentage decrease of iPTH. A cut-of value of 50% decrease of iPTH compared to the preoperative value ws chosen to be included in the statistical models. Sensitivity analysis included cut-of values of 30% and 70% decrease of iPTH. A two-sided alpha of 0.05 was used in all analyses to define statistical significance. Statistical analyses were performed using Stata/BC version 16.1 (StataCorp LLC, College Station, TX).

## Results

### Baseline demographic, clinical, and biochemical characteristics

A total of 607 patients were included, female to male ratio was 5:1. Mean age of all patients was 55.6 years, 77.9% (*N* = 473) had an ASA-score of I-II (Table 1). Multinodular goiter was the most frequent indication for surgery (*N* = 382, 62.9%), followed by hyperthyroidism/Graves’ disease (*N* = 135, 22.2%), and known malignancy (*N* = 57, 9.4%). Mean preoperative iPTH level was 49.0 pg/ml with a mean level of 29.3 pg/ml at the end of surgery. On postoperative day one, 20.4% (*N* = 124) of patients had hypocalcemia, most of which were asymptomatic (77.4%, *N* = 96 out of 124).

**Table 1:** Baseline demographic, clinical, and biochemical characteristics

Patient characteristics	Total *N* = 607
Patient age at surgery, mean (SD)	55.6 (15.3)
Sex	
Female	506 (83.4%)
Male	101 (16.6%)
ASA-Score	
ASA I/II	473 (77.9%)
ASA III/IV	134 (22.1%)
Indication for surgery	
Goiter	382 (62.9%)
Malignancy	57 (9.4%)
Hyperthyroidism/Graves' disease	135 (22.2%)
Recurrent Goiter	18 (3.0%)
Thyroiditis	15 (2.5%)
Extent of surgery	
Total thyroidectomy (TT)	568 (93.6%)
TT + lymph node dissection	39 (6.4%)
iPTH preoperative, mean (SD)	49 (23.6)
Re-operation	7 (1.2%)
Length of hospital stay in days, mean (SD)	2.6 (1.3)
iPTH postoperative, mean (SD)	29.3 (24.3)
Percentage decrease of iPTH, mean (SD)	−35.6 (47.2)
Postoperative hypocalcemia	124 (20.4%)
Symptomatic hypocalcemia	28 (4.6%)

### Univariable analysis

In univariable analysis, the proportion of patients undergoing total thyroidectomy with lymph node excision was higher in the group with low iPTH at the end of surgery of < 10 pg/ml compared to the group with iPTH of 10–15 pg/ml and > 15 pg/ml (13.3% vs. 4.9% vs. 3.9%, *p* < 0.01) (Table 2). Mean percentage of decrease of iPTH was highest among those patients with low iPTH of < 10 pg/ml (-76.9% vs. 63.9% vs. 18.3%, *p* < 0.01), this group of patients also had the highest rate of postoperative hypocalcemia on day one (45.0% vs. 26.0% vs 12.2%, *p* < 0.01). No statistical difference was found among group 1, 2 and 3 in terms of age, sex, ASA-Score and indication of surgery.

**Table 2:** Demographic, clinical, and biochemical characteristics according to postoperative iPTH-levels

	iPTH > 15 pg/ml *N* = 410	iPTH 10–15 pg/ml *N* = 77	iPTH < 10 pg/ml *N* = 120	*P* value*
Patient Characteristics				
Patient age at surgery, mean (SD)	56.6 (14.7)	53.4 (17.3)	53.4 (15.6)	0.05
Sex				< 0.01
Female	326 (79.5%)	72 (93.5%)	108 (90.0%)	
Male	84 (20.5%)	5 (6.5%)	12 (10.0%)	
ASA-Score				0.41
ASA I/II	320 (78.0%)	56 (72.7%)	97 (80.8%)	
ASA III/IV	90 (22.0%)	21 (27.3%)	23 (19.2%)	
Surgeon volume				0.44
Low volume surgeon	91 (22.2%)	21 (27.3%)	32 (26.7%)	
High volume thyroid surgeon	319 (77.8%)	56 (72.7%)	88 (73.3%)	
Indication for surgery				0.21
Goiter	255 (62.2%)	54 (70.1%)	73 (60.8%)	
Malignancy	35 (8.5%)	4 (5.2%)	18 (15.0%)	
Hyperthyroidism/Graves' disease	99 (24.1%)	14 (18.2%)	22 (18.3%)	
Recurrent Goiter	11 (2.7%)	4 (5.2%)	3 (2.5%)	
Thyroiditis	10 (2.4%)	1 (1.3%)	4 (3.3%)	
Extent of surgery				< 0.01
Total thyroidectomy (TT)	390 (95.1%)	74 (96.1%)	104 (86.7%)	
TT + lymph node dissection	20 (4.9%)	3 (3.9%)	16 (13.3%)	
iPTH preoperative, mean (SD)	51.9 (23.2)	46.1 (28.9)	40.7 (18.7)	<0.01
Re-operation	3 (0.7%)	2 (2.6%)	2 (1.7%)	0.31
Length of hospital stay in days, mean (SD)	2.6 (1.1)	2.7 (1.8)	2.8 (1.6)	0.18
iPTH postoperative, mean (SD)	39.3 (23.8)	12.6 (1.5)	6.2 (1.8)	<0.01
Percentage decrease of iPTH, mean (SD)	−18.3 (45.9)	-63.9 (20.9)	−76.9 (23.7)	<0.01
Decrease of iPTH postoperative				<0.01
iPTH-decrease 1–50%	229 (55.9%)	13 (16.9%)	2 (1.7%)	
iPTH-decrease > 50%	81 (19.8%)	62 (80.5%)	109 (90.8%)	
No decrease of PTH postoperativ	100 (24.4%)	2 (2.6%)	9 (7.5%)	
Postoperative Hypocalcemia	50 (12.2%)	20 (26.0%)	54 (45.0%)	<0.01
Symptomatic hypocalcemia	2 (0.5%)	4 (5.2%)	22 (18.3%)	<0.01

^*^ANOVA for continuous variables; chi-squared test for categorical variables

### Multivariable analysis – logistic regression

After multivariable adjustment, postoperative iPTH level and the percentage decrease of more than 50% compared to no decrease or decrease of 1–50% of iPTH were associated with postoperative hypocalcemia on postoperative day one (Table 3). In logistic regression model 1, iPTH of 10-15 pg/ml and < 10 pg/ml were associated with postoperative hypocalcemia (OR 2.29, *p* = 0.01; and OR 5.81, *p* < 0.01, respectively). While, in logistic regression model 2, patients that had a postoperative percentage decrease of iPTH of > 50% compared to 1–50% or no decrease were more likely to have hypocalcemia on day one after surgery (OR 7.08, *p* < 0.01). Finally, in model 3 which combined postoperative iPTH levels and percentage decrease of iPTH, iPTH levels of < 10 pg/ml compared to > 15 pg/ml (OR 2.01, *p* = 0.02) and percentage decrease of iPTH of > 50% compared to decrease of 1–50% or no decrease (OR 5.51, *p* < 0.01) were associated with hypocalcemia. The multivariable logistic regression model 3 which included iPTH levels and percentage decrease of iPTH had the lowest Akaike information criterion indicating the best statistical fit among the three models.

**Table 3:** Multivariable logistic regression models of characteristics associated with postoperative hypocalcemia

	Model 1	Model 2	Model 3
Variables	OR (95% CI); *p*-value	OR (95% CI); *p*-value	OR (95% CI); *p*-value
Patient age at surgery	0.99 (0.98–1.01); *p* = 0.27	0.99 (0.97–1.00); *p* = 0.17	0.99 (0.97–1.01); *p* = 0.19
Sex (Male vs. Female)	0.66 (0.34–1.27); *p* = 0.21	0.62 (0.32–1.22); *p* = 0.17	0.66 (0.34–1.29); *p* = 0.23
ASA Score (Ref. ASA I/II)			
ASA III/IV	1.49 (0.88–2.52); *p* = 0.14	1.50 (0.87–2.57); *p* = 0.14	1.60 (0.92–2.76); *p* = 0.10
Surgeon volume (high vs. low volume)	1.28 (0.77–2.13); *p* = 0.34	1.08 (0.64–1.81); *p* = 0.78	1.14 (0.67–1.93); *p* = 0.63
Indication (Ref. Goiter)			
Malignancy	0.93 (0.36–2.4); *p* = 0.89	0.76 (0.29–2.04); *p* = 0.59	0.75 (0.27–2.04); *p* = 0.57
Hyperthyroidism/Graves' disease	0.90 (0.52–1.57); *p* = 0.72	0.99 (0.56–1.75); *p* = 0.97	0.99 (0.55–1.75); *p* = 0.96
Recurrent Goiter	1.02 (0.3–3.47); *p* = 0.98	0.82 (0.24–2.79); *p* = 0.75	0.87 (0.25–3.04); *p* = 0.82
Thyroiditis	0.19 (0.02–1.57); *p* = 0.12	0.22 (0.03–1.81); *p* = 0.16	0.20 (0.02–1.65); *p* = 0.13
Extent of surgery (Ref. Total thyroidectomy)			
TT + lymph node dissection	1.08 (0.37–3.15); *p* = 0.89	1.48 (0.49–4.46); *p* = 0.49	1.27 (0.41–3.91); 0.68
iPTH-levels (Ref. > 15 pg/ml)			
iPTH 10.0—15.0 pg/ml	2.29 (1.25–4.18); *p* = 0.01		0.87 (0.44–1.72); *p* = 0.68
iPTH < 10.0 pg/ml	5.81 (3.59–9.41); *p* < 0.01		2.01 (1.12–3.59); *p* = 0.02
Percentage decrease of iPTH (Ref. decrease of 1–50% or no decrease)			
iPTH-decrease > 50%		7.08 (4.42–11.33); *p* < 0.01	5.51 (3.11–9.77); *p* < 0.01
Statistical model performance, AIC	573.1	544.3	539.7

*AIC* Akaike information criterion

### Sensitivity analysis

Sensitivity analyses with the cut-off value of 30% decrease of iPTH did not show an association with postoperative hypocalcemia. A relative reduction of iPTH of 70% was associated with a higher risk of postoperative hypocalcemia. However, the logistic regression model including 70% decrease of iPTH and iPTH levels did only show statistically significant associations for the relative reduction but not the iPTH value suggesting strong collinearity.

## Discussion

Hypocalcemia represents the most common complication following thyroid surgery and its early accurate identification and prediction allow for prompt intervention and management, reducing the risk of potentially life-threatening consequences such as tetany, seizures, laryngospasms and cardiac arrhythmias. Postoperative serum iPTH measurement serves as a valuable tool in predicting the likelihood of hypocalcemia following total thyroidectomy. However, variations of postoperative measurement may lead to difficulties in the interpretation of results and inconsistent clinical management regarding calcium and vitamin D supplementation.

Several studies have tried to evaluate the most appropriate time point to measure serum iPTH levels to accurately predict postoperative hypocalcemia. In studies of different research groups, iPTH levels have been measured during the operation, immediately after surgery, and/or at subsequent time intervals, that range from minutes to hours or even days after the procedure. Nagel et al. performed a meta-analysis of 188 studies and found that measurements of iPTH levels between the end of the operation and 24 h after surgery have a higher sensitivity and specificity in predicting hypocalcemia than the intraoperative values [22]. In the present study, serum iPTH levels were measured at the end of the operation, while the patient was still under general anesthesia. The results of this study are in line with the findings of another prospective study of Lang et al. that measured iPTH levels at the time of skin closure of 117 patients after total thyroidectomy or completion thyroidectomy [23]. This approach leads to the earliest possible detection of postoperative hypoparathyroidism and therefore allows for prompt calcium supplementation in case of reduced iPTH levels. Moreover, taking blood samples from patients under general anesthesia is more convenient for patients and staff compared to blood tests for example in the recovery room or on the surgical ward.

Early prediction of postoperative hypocalcemia is critical to prevent potentially life-threatening symptoms such as laryngospasm, muscle spasms, or cardiac arrhythmia [17]. Given the challenges of cross-referencing PTH tests, the relative reduction between pre- and postoperative PTH levels has been suggested as a suitable adjunct to predict postsurgical hypocalcemia[24]. According to the meta-analysis by Nagel et al. a mean reduction of PTH of 73% (SD 11%) was associated with postoperative hypocalcemia whereas patients with a mean PTH reduction of 39.5% (SD 7.3%) were at no risk of hypocalcemia [22]. After multivariable adjustment, the iPTH decrease of 50% or more was associated with postoperative hypocalcemia. The multivariable logistic regression model that included the relative reduction of iPTH together with iPTH-levels at the end of surgery had the best statistical fit compared to the model with either relative reduction or iPTH-levels alone suggesting a more accurate prediction of hypocalcemia for the combined model. The percentage decrease of iPTH levels should be used with caution due to inaccuracies of laboratory tests and other physiological factors, including vitamin D status. However, because of measurement inaccuracies and physiologic factors that influence iPTH levels, the combination of iPTH and relative iPTH reduction may provide a more accurate indication of the risk of postoperative hypocalcemia.

It is interesting to note that our findings regarding postoperative hypocalcemia, both in terms of biochemical and symptomatic, differ from what has been reported in previous research. In particular, a meta-analysis conducted by Sanabria et al., which included 11 studies, revealed that the rate of symptomatic hypocalcemia was 16.4%, while the biochemical hypocalcemia rate was 31.4% [25]. In contrast, the results of our study demonstrated significantly lower rates, 15.8% patients had asymptomatic, biochemical hypocalcemia and only 4.6% symptomatic hypocalcemia. This variation may be attributed to our approach of routinely administering calcium supplementation after surgery, particularly in cases where the levels of serum intact parathyroid hormone (iPTH) were low. This strategy has proven to be effective in reducing the incidence of hypocalcemia. By taking a proactive approach, we ensure that patients experience fewer or at least less severe postoperative symptoms associated with low iPTH levels. Ultimately, this approach improves patients' overall well-being and promotes faster recovery.

The results of our study contribute to the controversial issue whether Graves' disease is associated with a higher risk of postoperative hypocalcemia following total thyroidectomy. Several studies have shown that the increased vascularity of the thyroid glands and the autoimmune aspect of this disease could lead to a more challenging surgery and consequently higher risk for postoperative hypocalcemia [26, 27]. On the other hand, a meta-analysis of 23 studies by Chen et al. in 2021 demonstrated that Graves’ disease does not increase the risk for postoperative hypocalcaemia [28]. Similarly, our study showed that Graves’ disease was not significantly associated with the risk of postoperative hypocalcaemia neither in the uni- nor in the multivariable analysis.

This study has a few limitations. First, this was a retrospective study. However, the data was collected prospectively and the time of measurements of the serum iPTH and calcium was standardized. Second, the data were also gathered from a single institution, raising the possibility of institutional bias. Finally, since the postoperative serum iPTH determined calcium and vitamin D supplementation, it is possible that some of these patients may have received supplementation therapy to prevent symptomatic hypocalcemia even if it was not necessary.

## Conclusion

Standardizing an iPTH-protocol enhances patient care, improves the accuracy of postoperative assessment, and facilitates effective management of parathyroid dysfunction after total thyroidectomy. The combination of iPTH measurement at the end of surgery and relative reduction of iPTH is a cost-effective way to predict hypocalcemia. Timing of iPTH measurement at the end of total thyroidectomy is the earliest moment to detect patients at risk for postoperative hypocalcemia allowing for early calcium supplementation in those patients to avoid symptoms.

## Data Availability

The datasets used and/or analyzed during the current study are available from the corresponding author on reasonable request.

## Abbreviations

iPTH: Intact parathyroid hormone
ANOVA: Analysis of Variance
TT: Total thyroidectomy
ATA: American Thyroid Association
ETA: European Thyroid Association
DTC: Differentiated thyroid cancer
1,25(OH)2D: 1,25-Dihydroxyvitamin D
UTC: Undifferentiated thyroid carcinoma
NIFTP: Papillary-like nuclear features
EKBB: Ethikkommission Nordwest- und Zentralschweiz
RLN: Recurrent laryngeal nerve
ASA: American Society of Anesthesia
SD: Standard deviation
OR: Odds ratio
